# A systematic review and meta-analysis of Harmonic Focus in thyroidectomy compared to conventional techniques

**DOI:** 10.1186/s13044-015-0027-1

**Published:** 2015-10-01

**Authors:** Hang Cheng, Ireena Soleas, Nicole C. Ferko, Jeffrey W. Clymer, Joseph F. Amaral

**Affiliations:** Ethicon Inc, 4545 Creek Rd, Cincinnati, OH 45242 USA; Cornerstone Research Group, 204-3228 South Service Road, Burlington, ON L7N 3H8 Canada

**Keywords:** Harmonic Focus, Ultrasonic, Meta-analysis, Thyroidectomy

## Abstract

**Introduction:**

Several meta-analyses have been performed comparing the use of a variety of ultrasonic devices in thyroidectomy to conventional procedures. These studies have shown the superiority of ultrasonic devices for most outcomes studied including faster operative time and less blood loss, and equivalent or better safety for recurrent laryngeal nerve paresis and hypocalcemia. The current work is the first to examine a single ultrasonic device specifically designed for thyroid surgery, the Harmonic Focus, in order to confirm its efficacy and safety in thyroidectomy.

**Methods:**

A comprehensive literature search without language restrictions was performed for randomized clinical trials comparing Harmonic Focus and conventional clamp, cut and tie in thyroidectomy. Outcome measures included operating time, blood loss, post-operative pain, length of hospital stay, hypocalcemia and recurrent laryngeal nerve paresis. Risk of bias was analyzed for all studies. Meta-analysis was performed using random effects models with the inverse-variance method for mean differences of continuous variables and the Mantel-Haenszel method for risk ratios of dichotomous variables.

**Results:**

A total of 14 studies met the inclusion criteria. Harmonic Focus reduced operative time by 29 min, a 31 % decrease (*p* < 0.001), intra-operative blood loss by 45 ml (*p* < 0.001), post-operative pain (*p* < 0.001), length of hospital stay by 0.68 days (*p* = 0.005), drainage volume by 29 ml (*p* = 0.01), and occurrence of transient hypocalcemia by 40 % (*p* = 0.001). There were no significant differences between Harmonic Focus and conventional procedures in rate of persistent hypocalcemia, or rates of transient and persistent recurrent laryngeal nerve paresis.

**Conclusion:**

This is the first meta-analysis of Harmonic Focus in thyroid surgery. In agreement with meta-analyses previously performed on ultrasonic devices, use of the Harmonic Focus has been shown to be a more effective surgical procedure compared to conventional methods in thyroidectomy. The low occurrence of hypocalcemia and recurrent laryngeal nerve paresis confirms that Harmonic Focus can improve thyroidectomy efficiency without increasing the risk of complications.

## Introduction

Ultrasonic cutting and coagulation of soft tissues was introduced in 1991 with a primary focus on laparoscopic surgery in general and cholecystectomy in particular [1, 2]. There then followed a rapid evolution and adoption of the technology for vessel sealing that resulted in widespread use in a variety of intra-abdominal procedures including colectomy [3], fundoplication [4], and hysterectomy [5]. By the late 1990s and early 2000’s, endocrine and head and neck surgeons had adopted such devices for thyroid surgery noting decreased operating times [6]. Since then numerous randomized control trials (RCTs), large case series and numerous meta-analyses have been published confirming the reduction in operating time [7]. Furthermore, they have shown less blood loss (albeit not clinically significant), smaller incisions, less postoperative pain, reduced hospitalization and less drainage. Perhaps most importantly, studies that have evaluated the economic impact of using ultrasonic energy have concluded that there is overall cost savings when using such devices [8, 9].

A drawback of these studies is that they have used a variety of ultrasonic devices including Ultracision, Ace and Focus. However, since its introduction in 2007, the only specifically designed and approved device for use in thyroid and head and neck surgery is Harmonic Focus® (Ethicon Inc., Cincinnati OH). No meta-analysis or systematic review has been published to date evaluating specifically the impact of Harmonic Focus on the outcomes of thyroid surgery. Furthermore, some controversy exists in the literature concerning the impact of ultrasonic devices in general and Harmonic Focus in particular on parathyroid gland and recurrent laryngeal nerve function when these devices are used.

The purpose of this systematic review and meta-analysis is to answer two questions. First, what is the impact of Harmonic Focus on overall thyroid surgery outcomes when compared to the most commonly used clamp, cut and tie method that includes monopolar and or bipolar electrosurgery? Second, what impact does Harmonic Focus have on parathyroid and recurrent laryngeal nerve function following thyroid surgery?

## Methods

Twenty-one databases were systematically searched, including MEDLINE *via* PubMed, EMBASE, the Cochrane Central Register of Controlled Trials (CENTRAL), and 18 other national databases (Table 1). Reference lists of retrieved articles were reviewed and additional comprehensive searches were conducted through Google Scholar and Research-Gate. Publications of all languages were considered in the systematic review.

**Table 1:** List of databases and search periods included in systematic search

Databases	Search dates
EMBASE	Until 30th September 2014
MEDLINE (*via* PubMed)	
CENTRAL	
LILACS IBECS	Conducted between 26th and 30th September 2013
African Index Medicus, Index Medicus for Eastern and Mediterranean Region, Index Medcus for South-East Asia Region and The Western Pacific Region Index Medicus	
African Journals Online	
IndMed (India)	
PakMediNet (Pakistan)	
Türk Tip Veri Tabani (Turkey)	
Krack (Croatia)	
SID and IrMedex (Iran)	
KoreaMed (Korea)	
ICHUSHI-web (Japan)	Until 22nd April 2013
Wanfang, Cqvip, CNKI (China)	Until 16th April 2013

The PICOS categories (i.e., population, intervention, comparator, outcomes, and study design) were used to define study inclusion criteria. All published RCTs comparing the use of Harmonic surgical devices to conventional methods, such as monopolar or bipolar electrosurgery and suture, clips, or knot-tying in human subjects, for all surgery types, were considered for inclusion (Table 2). Benign and malignant disease were included if no lymph node dissection was performed. Full-text papers were excluded if, they were not a RCT, the principal surgical procedure was not thyroidectomy, devices other than the Harmonic Focus were used, and if lymphadenectomy, or only partial thyroidectomy, was conducted. The eligibility of each publication was evaluated by two independent reviewers (IS, NCF) and a third reviewer (HC) was consulted in the case of disagreements regarding study inclusion. When necessary, study authors were contacted for additional methodological details to confirm whether the study was randomized.

**Table 2:** Study and baseline characteristics for studies meeting inclusion criteria for open total thyroidectomy

Reference	Country	Interventions evaluated	n	Mean Age ± SD or (range)	% Male	Study length (months)	Included endpoints^a,^ ^b^
Askar 2011 [19]	Turkey	Harmonic Focus	65	41.81 ± 13.4	16.9 %	24	Operating time, Intra-operative blood loss, Length of stay, Post-operative pain, Drainage fluid volume, Hemorrhage requiring surgery, Hypocalcemia, RLN paresis
		Knot-tying with electrocautery	65	36.24 ± 12.62	24.6 %		
Cannizzaro 2014 [12]	Italy	Harmonic Focus	141	53 (12 to 81)	16.0 %	24	Operative time, Intra-operative blood loss, Length of stay, Serum calcium, Hypocalcemia, RLN paresis
		Monopolar or bipolar electrocautery, suture and clips	124				
Di Renzo 2010 [18]	Italy	Harmonic Focus	31	50.5 ± 12.1	26.0 %	8	Operating time, Length of stay, Hemorrhage requiring surgery, Hypocalcemia, RLN paresis
		Classic suture ligation	31	51.5 ± 13.7	22.0 %		
Docimo 2012 [16]	Italy	Harmonic Focus	100	46 (16 to 70)	30.0 %	24	Operating time, Hemorrhage requiring surgery, Hypocalcemia, RLN paresis, Wound seroma
		Conventional clamp and tie	100		40.0 %		
Duan 2013 [14]	China	Harmonic Focus	389	48.5 ± 21.8	4.89 %	Not reported	Operating time, Length of stay, Hemorrhage requiring surgery, RLN paresis, Hypocalcemia
		Suture/clip ligation withelectrocautery	389	50.1 ± 19.3	5.82 %		
Ferri 2011 [20]	Italy	Harmonic Focus	50	48.7 (21 to 73)	44.0 %	12	Operating time, Post-operative pain, Length of stay, Drainage fluid volume, Hypocalcemia, RLN paresis
		Knot tying with electrocautery	50	51.4 (23 to 72)	38.0 %		
Gentileschi 2011 [21]	Italy	Harmonic Focus	43	49.0 ± 13	20.9 %	12	Operating time, Length of stay, Hemorrhage requiring surgery, Hypocalcemia, RLN paresis
		Conventional technique (knot-tying with electrocautery)	38	48.0 ± 15	10.5 %		
Konturek 2012 [9]	Poland	Harmonic Focus	41	41.1 ± 7.5	17.1 %	11	Operating time, Intra-operative blood loss, Length of stay, Hemorrhage requiring surgery, Hypocalcemia, RLN paresis, Wound seroma
		Bipolar electrocautery and clip	41	42.0 ± 7.5	19.5 %		
Materazzi 2013 [17]	Italy	Harmonic Focus	141	51.68 ± 12.2	19.5 %	55	Operating time, Length of stay, Hemorrhage requiring surgery, Hypocalcemia, RLN paresis
		Conventional clamp and tie	127	53.97 ± 12.5	27.6 %		
Miccoli 2010 [10]	Italy	Harmonic Focus	31	48.6 (29 to 67)	29.0 %	Not reported	Operating time, Volume of drainage fluid, Post-operative pain, RLN paresis
		Suture/clip ligation with electrocautery	31	53.2 (18 to 75)	25.8 %		
Mourad 2011 [22]	Belgium	Harmonic Focus	34	50.0 ± 15	23.5 %	6	Operating time, Intra-operative blood loss, Hypocalcemia, RLN paresis
		Monopolar electrocautery with clamp and tie	34	47.0 ± 12	23.5 %		
Pons 2009 [8]	France	Harmonic Focus	20	55.0 ± 11	20.0 %	Not reported	Operating time, Intra-operative blood loss, Hemorrhage requiring surgery, Hypocalcemia, RLN paresis
		Conventional clamp and tie	20				
Sista 2012 [13]	Italy	Harmonic Focus	130	49.3 (32 to 76))	23.8 %	14	Operating time, RLN paresis, Hypocalcemia
		Monopolar or bipolar diathermy and ligature	131	51.1 (39 to 78)	22.1 %		
Soroush 2013 [15]	Iran	Harmonic Focus	33	38.7 ± 13.5	48.5 %	12	Operating time, Intra-operative blood loss, Length of stay, RLN paresis
		Conventional clamp and tie	35	43.2 ± 14.5	54.3 %		

^a^Length of stay endpoint refers to length of hospitalization stay

^b^Reported endpoints that did not separate total and partial thyroidectomy results were excluded from the analysis

Study details (i.e., baseline characteristics and outcomes) of included publications were extracted through a standardized data extraction form. Two reviewers extracted data independently and any inconsistencies were resolved by consensus or by consultation with a third reviewer. Non-English publications were translated and data extraction was completed. Data extraction by one reviewer was subsequently cross-checked by a second reviewer.

The following clinical outcome measures were included: (1) operating time, (2) intra-operative blood loss, (3) post-operative pain, (4) length of hospitalization, (5) volume of drainage fluid, (6) hemorrhage requiring surgery, (7) hypocalcemia (transient and persistent), (8) recurrent laryngeal nerve (RLN) paresis (transient and persistent), and (9) wound seroma. Inclusion criteria for the persistent RLN paresis parameter required at least six months follow-up unless RLN paresis was not reported prior to six months. RLN paresis needed to be determined *via* laryngoscopy. In studies where results were reported for transient RLN paresis, but not persistent RLN paresis, it was assumed that there were zero cases of the persistent type. The same assumption was applied to the transient and persistent hypocalcemia outcome. Both clinically- and chemically-determined hypocalcemia were included in the hypocalcemia parameter. The standard deviation (SD) variance measure was not reported in one study [10] for operating time. Study authors were not contacted to retrieve missing data, however, standard methods provided by Cochrane [11] were used to impute the missing variance measure in this study.

The Cochrane Collaboration tool [11] for assessing risk of bias was used to evaluate the quality of the included studies. Based on seven pre-specified domains (sequence generation, allocation concealment, blinding of participants and personnel, blinding of outcome assessment, incomplete outcome data, selective outcome reporting, and other sources of bias), publications were scored as having low, unclear, or high risk of bias. Final quality assessments were based on the combination of these factors and individual study characteristics. Two authors independently assessed the study quality and inconsistencies were resolved through consensus or by discussion with a third author.

Review Manager (Version 5.3, The Nordic Cochrane Centre, The Cochrane Collaboration, Copenhagen, Denmark, 2014) was used to perform the meta-analysis. The inverse-variance method was used to calculate the mean differences (MD) for continuous outcomes (operating time, intra-operative blood loss, post-operative pain, length of hospitalization, volume of drainage fluid). Risk ratios (RR) were calculated for dichotomous outcomes (hemorrhage requiring surgery, wound seroma, transient hypocalcemia, persistent hypocalcemia, transient RLN paresis, persistent RLN paresis) using the Mantel-Haenszel method. The meta-analysis used a random effects model and forest plots for all included outcomes were generated using Review Manager. Study heterogeneity was evaluated through the *χ*^2^ test and *I*^2^ measure.

The Harmonic Focus was compared to conventional methods in the primary analysis. Sensitivity analyses were conducted for the imputation of missing variance measures, where studies requiring imputation were excluded [10]. Further sensitivity analyses were completed for study quality, where studies with unclear or high risk of bias across several measures were excluded [8, 12–15]. Sensitivity analyses excluding studies that did not include electrocautery as part of the conventional technique [8, 15–18] were also performed.

## Results

**Fig. 1:**
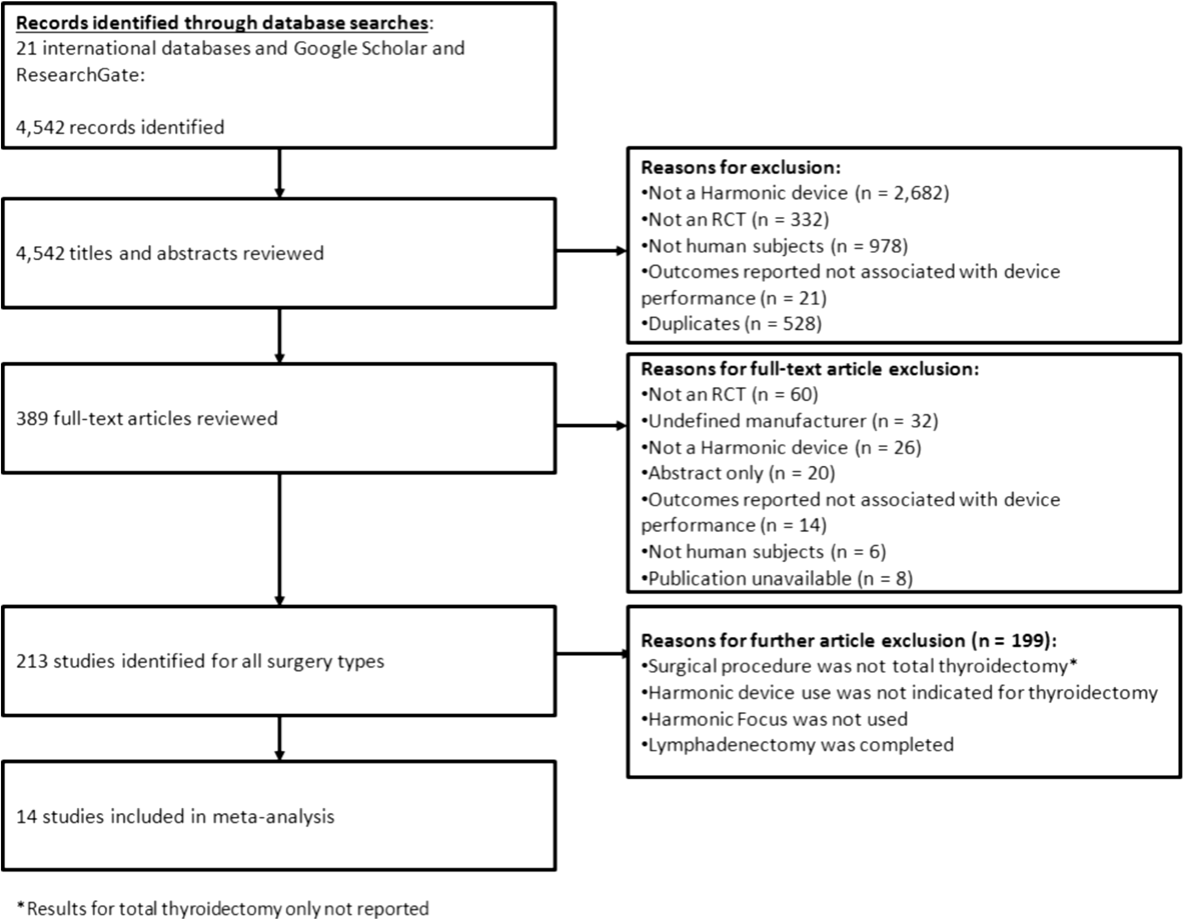
PRISMA diagram for the systematic literature review

The systematic search resulted in the identification of 4542 total records, of which 4153 were excluded following title and abstract screening (Fig. 1). Of the 389 full text articles retrieved and reviewed, 375 were further excluded if studies were non-RCTs, had an undefined manufacturer, did not use Harmonic Focus, the publication was unavailable and had nonhuman subjects, or the surgical procedure was not total thyroidectomy. Overall, 14 studies consisting of 2516 patients in total reporting on Harmonic device (Focus) use in total thyroidectomy were included in the meta-analysis [8–10, 12–22].

Study characteristics are summarized in Table 2. The included studies ranged in sample size from 40 to 778, and study length spanned from 6 to 55 months. In all studies, the Harmonic Focus surgical device was compared to conventional thyroidectomy techniques. In total, four studies [8, 15–17] compared the Focus to conventional clamp and tie; one study [18] compared to classic suture ligation; three studies [19–21] compared to knot tying with electrocautery; and six studies [9, 10, 12–14, 22] compared to monopolar or bipolar electrocautery with one or more of sutures, clips, or clamp and tie. Total and partial thyroidectomy was performed in one study [13]; however, outcome results for partial thyroidectomy were not included. Of the 14 included studies, the majority were European, with 8 studies from Italy alone. Studies typically reported a wide range of outcomes. All included studies assessed operating time [8–10, 12–22], while intra-operative blood loss was reported in six studies [8, 9, 12, 15, 19, 22]. Three studies assessed post-operative pain based on a visual analogue scale (VAS) [10, 19, 20], nine studies reported the length of hospitalization [9, 12, 14, 15, 17–21] and three studies reported on drainage volume [10, 19, 20]. Overall complication rate was not estimable as the two studies informing the analysis were assumed to have zero complications [9, 13]. Instead, separate outcomes were reported for hemorrhage requiring surgery [8, 9, 14, 16–19, 21], transient and persistent hypocalcemia [8, 9, 12–14, 16–22], transient and persistent RLN paresis [8–10, 12, 14–22], and wound seroma [9, 16]. Hypocalcemia was chemically-determined in 11 studies [8–10, 12, 14, 16–20, 22] and clinically-determined in two studies [13, 21]. One study did not specify whether hypocalcemia was chemically- or clinically-determined [15].

**Fig. 2:**
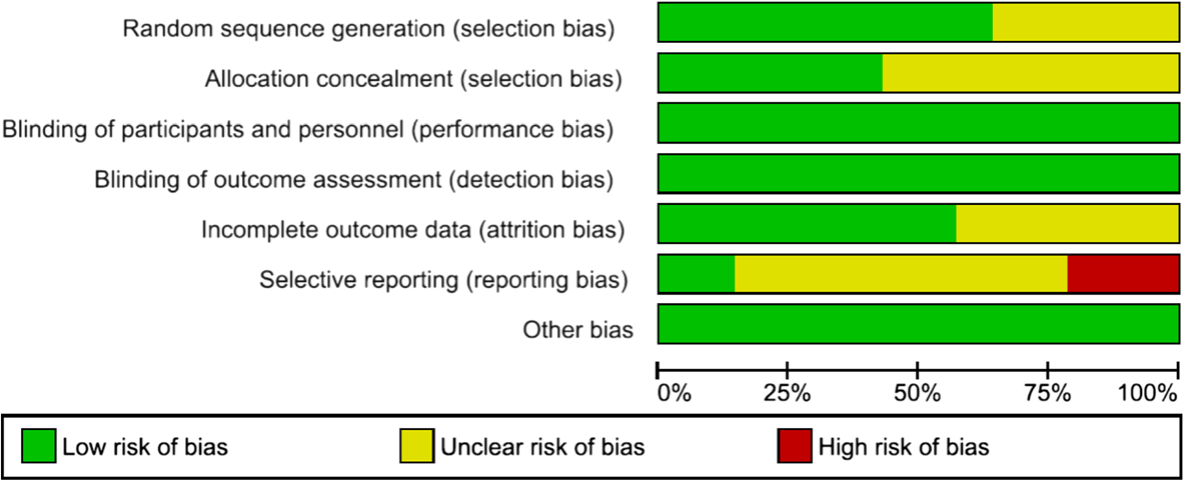
Risk of bias assessment for studies meeting inclusion criteria

The risk of bias varied across the included studies. The overall results of the risk of bias assessments are reported in Fig. 2 and individual study quality assessments are summarized in Table 3. Randomization method was known in nine studies [8–10, 12, 16, 17, 19–22]. Three studies described randomization through the use of envelopes [10, 12, 17], two used a random permuted block design [9, 22], and two described the use of a drawing technique [8, 21]. One study used a computer-generated schedule [19] and one described a fixed simple randomization method [20]. Six studies [9, 10, 12, 17, 19, 22] described concealment of the randomization sequence. Blinding of patients to the surgical technique was reported in six studies [8–10, 14, 15, 20], one study [22] reported blinding of the surgeon to the surgical technique, and three studies [9, 10, 19] described blinding of outcome assessors. Risk of performance bias was deemed low in non-blinded studies, as outcomes were considered objective and unlikely to be affected by a lack of blinding. There were no patient withdrawals in seven studies [8, 9, 12, 16–18, 20] and one study [22] reported exclusions, but was assumed to have no clinically relevant impact. Reporting of attritions or exclusions was insufficient in six studies [10, 13–15, 19, 21]. Selective reporting remained unclear in nine studies [9, 10, 14–18, 20, 21], while three studies were deemed to have a high risk of bias as certain outcomes noted in protocol were not included in the results [8, 12, 13].

**Table 3:** Qualitative risk of bias assessment summary

Study	Sequence generation	Allocation concealment	Blinding of personnel and participants	Blinding of outcomes	Incomplete outcome data addressed	Free of selective reporting	Free of other bias
Askar 2011 [19]	Yes	Yes	Yes	Yes	Unclear	Yes	Yes
Cannizzaro 2014 [12]	Yes	Yes	Yes	Yes	Yes	No	Yes
Di Renzo 2010 [18]	Unclear	Unclear	Yes	Yes	Yes	Unclear	Yes
Docimo 2012 [16]	Unclear	Unclear	Yes	Yes	Yes	Unclear	Yes
Duan 2013 [14]	Unclear	Unclear	Yes	Yes	Unclear	Unclear	Yes
Ferri 2011 [20]	Yes	Unclear	Yes	Yes	Yes	Unclear	Yes
Gentileschi 2011 [21]	Yes	Unclear	Yes	Yes	Unclear	Unclear	Yes
Konturek 2012 [9]	Yes	Yes	Yes	Yes	Yes	Unclear	Yes
Materazzi 2013 [17]	Yes	Yes	Yes	Yes	Yes	Unclear	Yes
Miccoli 2010 [10]	Yes	Yes	Yes	Yes	Unclear	Unclear	Yes
Mourad 2011 [22]	Yes	Yes	Yes	Yes	Yes	Yes	Yes
Pons 2009 [8]	Yes	Unclear	Yes	Yes	Yes	No	Yes
Sista 2012 [13]	Unclear	Unclear	Yes	Yes	Unclear	No	Yes
Soroush 2013 [15]	Unclear	Unclear	Yes	Yes	Unclear	Unclear	Yes

*Yes* low risk of bias, *No* high risk of bias

### Operating time

**Fig. 3:**
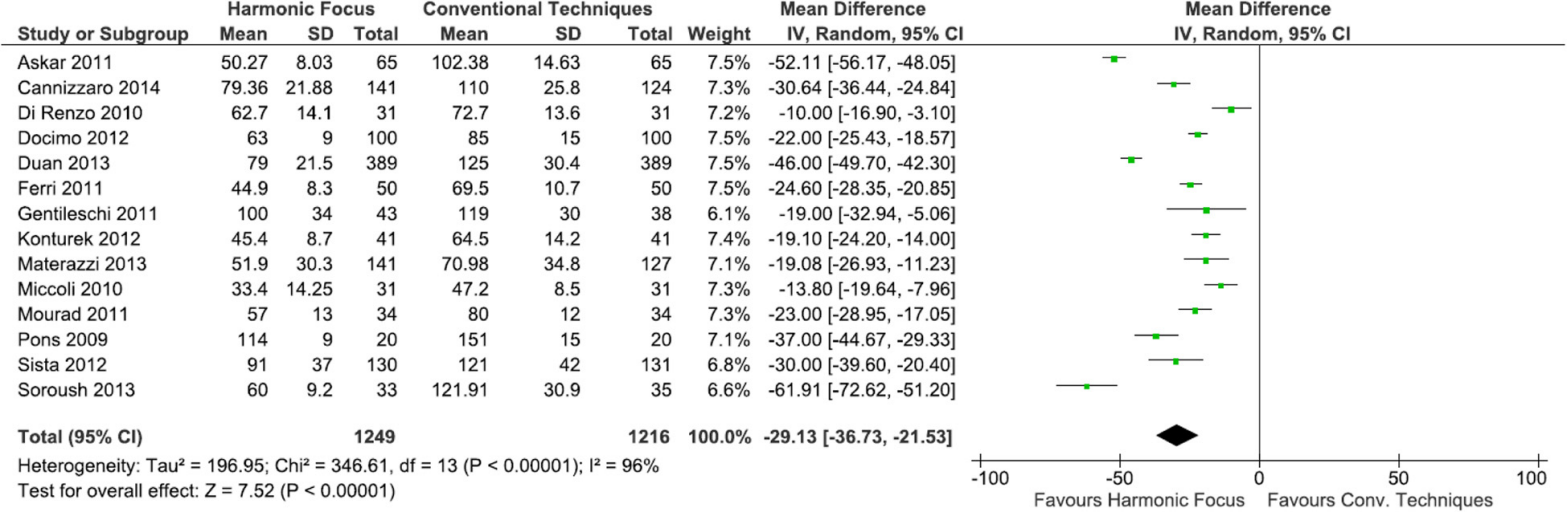
Forest plot of meta-analysis results for operating time (minutes)

Mean operating time (Harmonic Focus: 66.08 min, conventional technique: 95.26 min) was statistically significantly reduced by 29.13 min (95 % CI: −36.73 to −21.53; *P* < 0.00001; 14 studies; *I*^2^ = 96 %), a 30.6 % decrease with the Harmonic Focus compared to conventional methods in total thyroidectomy (Fig. 3).

### Intra-operative blood loss

**Fig. 4:**
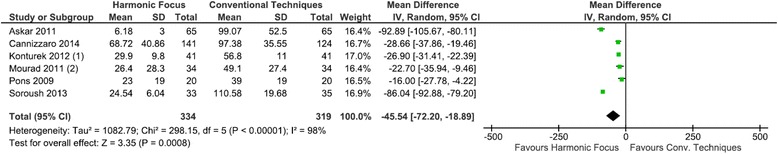
Forest plot of meta-analysis results for intra-operative blood loss (mL)

Mean intra-operative blood loss (Harmonic Focus: 29.84 mL, conventional technique: 75.34 mL) was statistically significantly reduced by 45.54 mL (95 % CI: −72.20 to −18.89; *P* = 0.0008; 6 studies; *I*^2^ = 98 %), a 60.4 % decrease with the Harmonic Focus compared to conventional methods in total thyroidectomy (Fig. 4).

### Post-operative pain

**Fig. 5:**
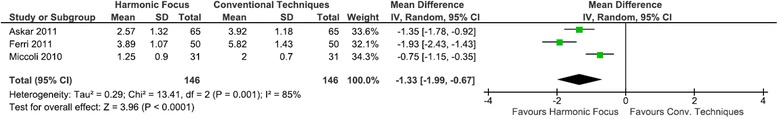
Forest plot of meta-analysis results for post-operative pain (VAS)

On the basis of three studies comparing the Harmonic Focus to conventional techniques in total thyroidectomy, a statistically significant reduction in mean VAS reported post-operative pain (Harmonic Focus: 2.54, conventional technique: 3.87) by 1.33 points (95 % CI: −1.99 to −0.67; *P* < 0.0001; 3 studies; *I*^*2*^ = 85 %) was observed with the Harmonic Focus (Fig. 5).

### Length of hospital stay

**Fig. 6:**
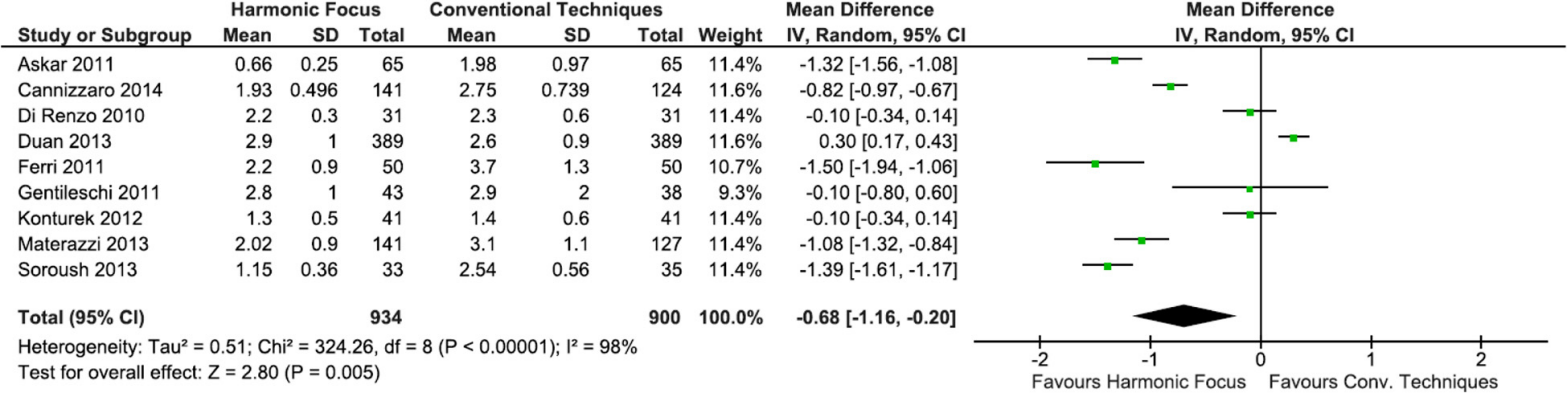
Forest plot of meta-analysis results for length of hospitalization stay (days)

Results demonstrated a statistically significant reduction of 0.68 days in the mean postoperative length of hospitalization (Harmonic Focus: 1.89 days, conventional technique: 2.58 days) with the Harmonic Focus (95 % CI: −1.16 to −0.20; *P* = 0.005; 9 studies; *I*^*2*^ = 98 %), a 26.4 % decrease, compared to conventional techniques in total thyroidectomy (Fig. 6).

### Drainage volume

**Fig. 7:**
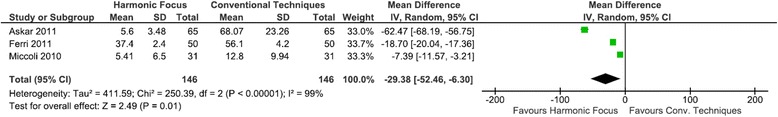
Forest plot of meta-analysis results for volume of drainage fluid (mL)

In contrast to conventional methods in total thyroidectomy, mean drainage volume (Harmonic Focus: 16.25 mL, conventional technique: 45.63 mL) was statistically significantly reduced by 29.38 mL (95 % CI: −52.46 to −6.30; *P* = 0.01; 3 studies; *I*^*2*^ = 99 %) with the Harmonic Focus (Fig. 7).

### Hemorrhage requiring surgery

**Fig. 8:**
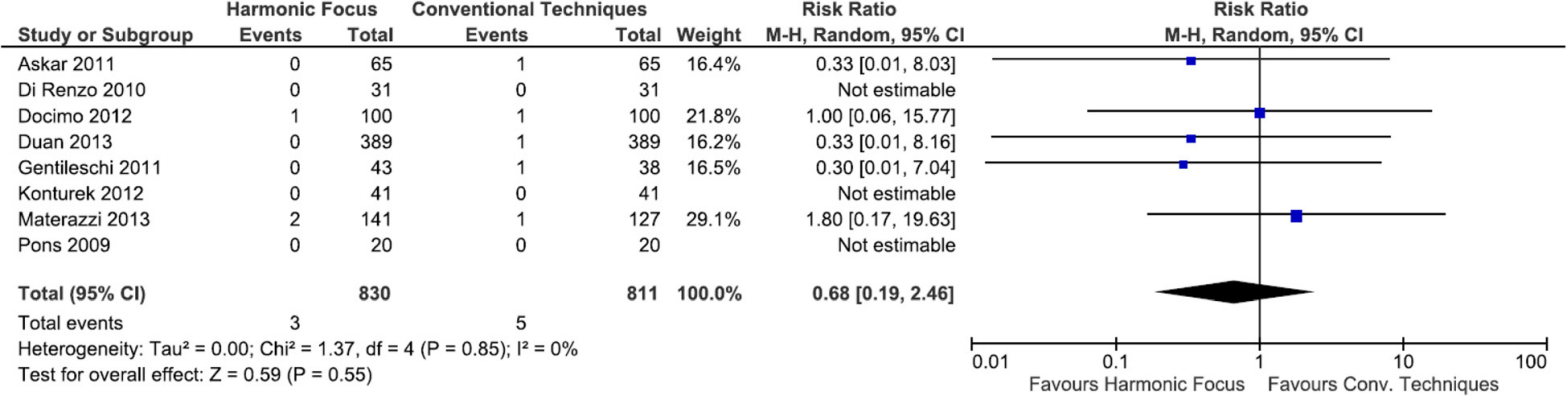
Forest plot of meta-analysis results for hemorrhage requiring surgery

Five of the eight studies examining post-operative bleeding requiring re-operation reported on hemorrhage events. Results demonstrated no statistically significant difference in bleeding events requiring re-operation between the Harmonic Focus and conventional methods (RR = 0.68; 95 % CI: 0.19 to 2.46; *P* = 0.55; 8 studies; *I*^*2*^ = 0 %) (Fig. 8).

### Hypocalcemia

**Fig. 9:**
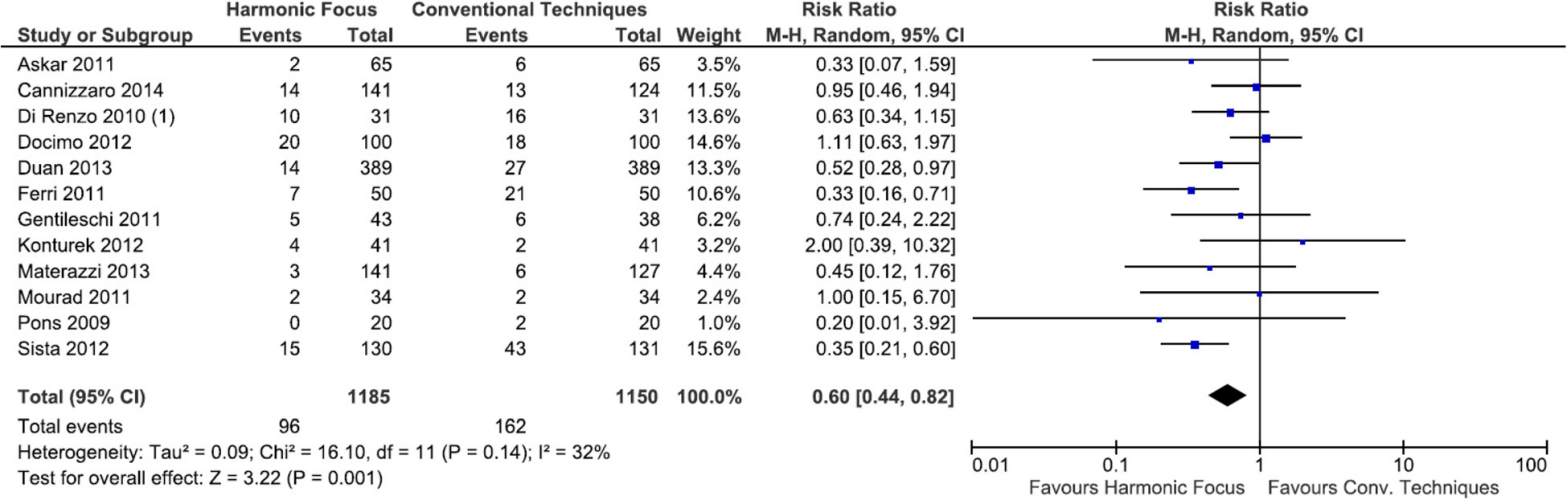
Forest plot of meta-analysis results for transient hypocalcemia

**Fig. 10:**
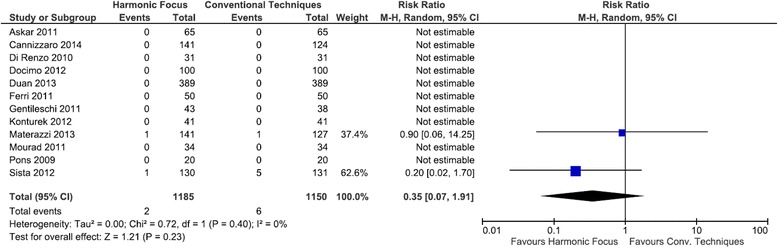
Forest plot of meta-analysis results for persistent hypocalcemia

Compared to conventional techniques in total thyroidectomy, the Harmonic Focus resulted in a statistically significant reduction in transient hypocalcemia with a RR of 0.60 (95 % CI: 0.44 to 0.82; *P* = 0.001; 12 studies; *I*^*2*^ = 32 %) (Fig. 9). Persistent hypocalcemia events were reported in only two of the twelve studies examining this outcome. Results demonstrated a lower risk of persistent hypocalcemia with the Harmonic Focus than with conventional methods, although not statistically significant (RR = 0.35; 95 % CI: 0.07 to 1.91; *P* = 0.23; 12 studies; *I*^*2*^ = 0 %) (Fig. 10).

### RLN paresis

**Fig. 11:**
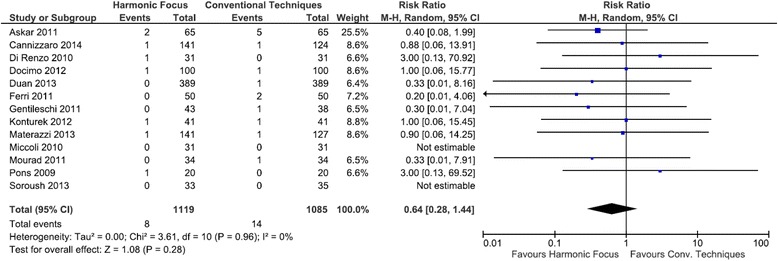
Forest plot of meta-analysis results for transient RLN paresis

Harmonic Focus use was associated with fewer transient RLN paresis events compared with conventional thyroidectomy techniques, but these results were not statistically significant (RR = 0.64; 95 % CI: 0.28 to 1.44; *P* = 0.28; 13 studies; *I*^*2*^ = 0 %) (Fig. 11). Of the thirteen studies examining persistent RLN paresis, only one study reported an event. In this study, the Harmonic Focus reduced the risk of persistent RLN paresis, although results were not statistically significant (RR = 0.33; 95 % CI: 0.01 to 8.03; *P* = 0.50) (Figure not shown).

### Wound seroma

**Fig. 12:**
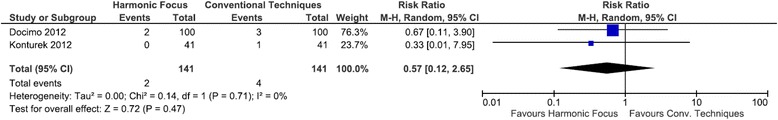
Forest plot of meta-analysis results for wound seroma

No significant differences were reported between Harmonic Focus and conventional methods for the outcome of wound seroma in total thyroidectomy (RR = 0.57; 95 % CI: 0.12 to 2.65; *P* = 0.47; 2 studies; *I*^*2*^ = 0 %) (Fig. 12).

### Sensitivity analyses

Sensitivity analyses demonstrated results similar to the primary analysis and were relatively robust to variables tested. Results for operating time, intraoperative blood loss, post-operative pain, length of hospitalization and transient hypocalcemia remained statistically significantly lower with the Harmonic Focus when studies with a higher risk of bias were excluded (Table 4). Additionally, primary analysis results were relatively robust to the exclusion of studies with conventional techniques that did not utilize monopolar or bipolar electrocautery or when imputed results by Miccoli et al. [10] were excluded for operating time.

**Table 4:** Summary of primary and sensitivity analyses

Outcome	Primary analysis	Sensitivity analyses		
		Excluding ‘lower’ quality studies [8, 12–15] ^a^	Excluding imputed data [10] ^b^	Excluding studies that did not use electrocautery in conventional [8, 15–18] ^c^
Operating Time (min)	−29.13 (−36.73, −21.53)	−22.72 (−31.75, −13.68)	−30.34 (−38.13, −22.54)	−28.95 (−38.44, −19.35)
(MD [95 %CI])				
Intraoperative Blood Loss (mL)	−45.54 (−72.20, −18.89)	−42.48 (−67.97, −16.99)	Identical to primary analysis	−42.48 (−67.97, −16.99)
(MD [95 %CI])				
Post-Operative Pain (VAS)	−1.33 (−1.99, −0.67)	−1.33 (−1.99, −0.67)		−1.33 (−1.99, −0.67)
(MD [95 %CI])				
Length of Stay (days)	−0.68 (−1.16, −0.20)	−0.71 (−1.24, −0.18)		−0.59 (−1.21, 0.02)
(MD [95 %CI])				
Drainage Volume (mL)	−29.38 (−52.46, −6.30)	−29.38 (−52.46, −6.30)		−29.38 (−52.46, −6.30)
(MD [95 %CI])				
Hemorrhage require surgery	0.68 (0.19, 2.46)	0.78 (0.19, 3.18)		0.32 (0.05, 2.01)
(RR [95 %CI])				
Transient Hypocalcemia	0.60 (0.44, 0.82)	0.67 (0.45, 1.00)		0.54 (0.37, 0.78)
(RR [95 %CI])				
Persistent Hypocalcemia	0.35 (0.07, 1.91)	0.90 (0.06, 14.25)		Too few studies to inform (<2)
(RR [95 %CI])				
Transient RLN Paresis	0.64 (0.28, 1.44)	0.57 (0.23, 1.43)		0.43 (0.16, 1.14)
(RR [95 %CI])				
Persistent RLN Paresis	0.33 (0.01, 8.03)	0.33 (0.01, 8.03)		Too few studies to inform (<2)
(RR [95 %CI])				
Wound Seroma	0.57 (0.12, 2.65)	0.57 (0.12, 2.65)		Too few studies to inform (<2)
(RR [95 %CI])				

*CI* Confidence Interval, *LOS* Length of Stay, *MD* Mean Difference, *RR* Relative Risk, *VAS* Visual Analog Scale, *min* minutes, *mL* milliliters

^a^Lower quality study defined as: ≥ 4 “unclear” OR one “No” listed in any risk of bias assessment category: Cannizzaro [12], Pons [8], Sista [13], Duan [14], Soroush [15]

^b^Miccoli [10]

^c^Di Renzo [14], Docimo [16], Materazzi [17], Pons [8], Soroush [15]

## Discussion

Although thyroidectomy was not one of the initial applications of ultrasonic surgery, it was realized that use of the Harmonic scalpel provides many advantages over the conventional procedures. Because of the high vascularization of the thyroid gland, efficient and meticulous hemostasis is required to reduce the risk of complications and post-operative morbidity. As with any surgical procedure, a reduction in operative time will typically lessen the chance of surgical-site infection and may lead to faster patient recovery time [23]. Rapid coagulation with the Harmonic Focus can substantially increase the speed of the operation. In addition, the precision of the Harmonic Focus can ensure that the risk of the two principal adverse events, namely hypocalcemia and recurrent laryngeal nerve paresis, are not elevated, or may even be reduced.

Five standard meta-analyses have compared ultrasonic devices to conventional techniques in thyroidectomy. These previous findings are in general similar to those in this study. In analyzing 9 studies, Melck [24] observed significantly faster operative time (−23.1 min, *p* < 0.001), and less transient hypocalcemia (RR 0.69, *p* = 0.01) for use of ultrasonic devices compared to conventional methods. In 7 studies, Cirocchi [25] found significantly faster operative time (−18.7 min, *p* < 0.001), less blood loss (−60.1 ml, *p* = 0.04), and less drainage volume (−35.3 ml, *p* < 0.001). In 11 studies, Zhang [26] found significantly faster operative time (−22.4 min, *p* < 0.001), and less intraoperative bleeding (−26.6 ml, *p* = 0.02). In 12 studies, Ecker [27] observed significantly faster operative time (−22.7 min, *p* < 0.001), less blood loss (−20.0 ml, *p* < 0.001), less post-operative pain (−0.86 units, *p* = 0.02), and reduced length of hospital stay (−0.12 days, *p* = 0.05). Finally, in 13 studies, Zhao [28] found significantly faster operative time (−21.1 min, *p* < 0.001), less intra-operative blood loss (−14.4 ml, *p* < 0.001), less drainage (−7.5 ml, *p* < 0.001), and lower hospitalization charges (−118 USD, *p* < 0.001). None of these meta-analyses found a higher rate of transient or persistent RLN paresis for ultrasonic devices compared to conventional techniques.

Recently two network meta-analyses have been performed. In these studies, ultrasonic devices were compared to both conventional techniques and advanced bipolar technology. In one of these network meta-analyses, Contin [7] evaluated 21 studies and found significantly faster operative time (−22.3 min, *p* < 0.001), less intraoperative blood loss (−28.5 ml, *p* < 0.001), shorter hospital stay (−0.28 days, *p* = 0.016) and less post-operative bleeding (−11.2 ml, *p* < 0.001) for ultrasonic devices. There was a trend toward lower transient hypocalcemia (*p* = 0.066) for ultrasonic, and importantly no difference in rates of transient RLN paresis (*p* = 0.847) or persistent RLN paresis (*p* = 0.711). Interestingly, the mean difference of operative time for investigator-initiated trials was greater than for industry-sponsored trials, belying the notion in this case that industry trials are necessarily biased toward their own commercial product.

In the other network meta-analysis, Garas [29] claimed to use 25 RCT’s, although one of the studies misidentified an electrosurgical device as ultrasonic and another study was not randomized [7, 30]. In comparing ultrasonic to conventional, they reported significantly faster operative time (*p* < 0.01), less blood loss (*p* < 0.01), less drain output (*p* = 0.03), and lower cost (*p* = 0.03), but a trend towards higher persistent RLN paresis (*p* = 0.08) for ultrasonic. A re-tabulation of the studies included in the Garas analysis [30] indicated 1/1006 (0.1 %) cases of persistent RLN paresis for ultrasonic *versus* 2/992 (0.2 %) for conventional surgery. In the current work, which included only studies where Harmonic Focus was used, there was no difference in the rate of persistent RLN paresis (*p* = 0.50), with zero cases among a total of 1119 subjects for Harmonic (0.0 %) and one case among 1085 subjects for conventional techniques (0.1 %). This lack of difference was also confirmed in the Contin network meta-analysis [7], which suggested that the results of Garas “should be read with caution.” Based on the results of the standard and network meta-analyses, including re-analysis of the Garas study, and the current study, all of which show no difference between ultrasonic devices and conventional techniques, there should be high confidence that Harmonic Focus can be used thyroidectomy with a low risk of RLN paresis, equivalent to conventional clamp, cut and tie.

In contrast to all previous efforts, the current study was limited to only Harmonic Focus as the ultrasonic device of interest. The Harmonic Focus is designed for use in open procedures and is specifically cleared for applications in head and neck surgery. A small end effector enables precise placement and the ultrasonic energy provides rapid dissection and coagulation. As evidenced in all studies to date, the design of the Harmonic Focus gives substantial advantages over conventional techniques in thyroidectomy.

In the current study, we observed operative time was significantly faster for Harmonic Focus compared to conventional clamp, cut, and tie. This speed increase is a result of both faster dissection and better hemostasis. Shorter operative time can lead to improved patient outcomes and faster recovery, and provide operative and overall hospital cost savings [8, 9]. The difference of 29 min observed in this study represents a 31 % decrease in operative time compared to conventional technique. Other measures related to the hemostasis capabilities are intra-operative blood loss and drainage volume, where use of Focus led to decreases of 60 % and 64 %, respectively, compared to clamp, cut and tie.

Post-operative pain was 34 % lower for Harmonic Focus than clamp, cut, and tie. This advantage may be related to shorter operative time noted above and observations that ultrasonic devices generally cause less inflammation [31–33]. The decrease of 1.33 points represents a 34 % reduction in pain compared to conventional surgery. The decrease in length of hospital stay, a 26 % reduction relative to conventional procedures, may be related to shorter operative time, lessened blood loss and drainage and less post-operative pain.

For the dichotomous results evaluated, namely, hemorrhage requiring surgery, hypocalcemia, RLN paresis, and wound seroma, all were less frequent with Harmonic than conventional technique, but because of the low frequency of all these events, only for transient hypocalcemia was there a significant difference, with Harmonic having a 40 % lower rate of occurrence than conventional clamp, cut, and tie. This significantly lower rate of transient hypocalcemia may be a result of the shorter operative time and reduced overall systemic impact of surgery with Harmonic Focus compared to conventional surgery. A drop in calcium blood levels may result not just from unintended damage to the parathyroid gland by clamp, cut and tie, but also be a typical systemic response to longer, more involved surgical procedures. While Harmonic Focus provides a lower rate of transient hypocalcemia, there does not appear to be a long-term difference, as the rate of persistent hypocalcemia is not significantly different from conventional procedures.

As demonstrated in this and all other reliable meta-analyses, the rate of both transient and persistent RLN paresis with ultrasonic devices is not greater than with clamp, cut & tie procedures. In fact, the trend with Harmonic Focus suggests that with more data it may be possible to show a reduction in transient RLN paresis. The cause of transient RLN paresis is generally assigned to excess traction. Use of continuous intra-operative nerve monitoring with stimulation to the vagus nerve can avoid paresis if the traction is reduced when the electromyographic reading has decreased by 50 % or more [34]. The improved dissecting ability of ultrasonic devices may decrease the amount of traction compared to clamp, cut and tie. The etiology of persistent paresis is typically prolonged thermal, electrical or direct contact with transection of the RLN. To avoid this complication, it is critical to visually identify the RLN during surgery. Use of an ultrasonic device eliminates electrical current and gives a wider margin of error for thermal effects compared to monopolar electrosurgery.

Numerous preclinical studies have evaluated the impact of Harmonic technology when used close to a nerve [32]. All studies to date have shown an effect on nerve function similar to that of cold steel scalpel when used up to 2 mm from the nerve. Important caveats to remember are that activation time should be less than 15 s and the nerve itself should never be touched directly either during activation or immediately afterwards. As all energy technologies generate heat, they must be carefully managed. In contrast to ultrasonic energy, monopolar and bipolar technologies produce at minimum transient injury when used up to 5 mm from the nerve. Recently, the potential adverse effects of advanced bipolar technologies were demonstrated with a doubling of β-amyloid precursor protein, a marker of impaired axonal transport, and a 133 % increase in neural inflammation when compared to ultrasonic energy [35].

Despite the comfort that can be developed from studies evaluating Focus, it must be stressed that no technology will replace meticulous surgical techniques and no technology is fool-proof. In that regard, the important elements must always be kept foremost in the surgeon’s mind when using Harmonic Focus. First, activations should occur as far from nerves or glands as possible, and no closer than 2 mm. Second, continuous activation should be less than 15 s as one approaches the nerve. Finally, the heat of the instrument must be actively managed by cooling, and an activated instrument should never touch the nerve during dissection.

## Conclusions

The Harmonic Focus is a more effective surgical device compared to conventional techniques in thyroidectomy. Its use offers several clinical advantages, including reduced operating time, intra-operative blood loss, drainage volume, post-operative pain, length of hospital stay, and transient hypocalcemia which can ultimately benefit the surgeon, patient and hospital, without the addition of safety concerns.

All studies performed to-date have used Harmonic Focus, prior to the introduction of an improved version, Harmonic Focus+, which has a substantially smaller end effector and produces a significantly smaller thermal effect [35]. All of the benefits observed in this study are expected to be maintained or even improved upon with the increased precision of the new device.
